# Genetic screening of regulatory regions of pituitary transcription factors in patients with idiopathic pituitary hormone deficiencies

**DOI:** 10.1007/s11102-017-0850-6

**Published:** 2017-12-18

**Authors:** Melitza Elizabeth, Anita C. S. Hokken-Koelega, Joyce Schuilwerve, Robin P. Peeters, Theo J. Visser, Laura C. G. de Graaff

**Affiliations:** 1 Dutch Growth Research Foundation, Rotterdam, The Netherlands; 2 Pediatrics, Subdivision Endocrinology, Erasmus MC Rotterdam, Rotterdam, The Netherlands; 3 Academic Center for Growth Disorders, Erasmus MC Rotterdam, Rotterdam, The Netherlands; 4 Internal Medicine, Subdivision Endocrinology, Erasmus MC Rotterdam, Rotterdam, The Netherlands; 5 Academic Center for Thyroid Diseases, Erasmus MC Rotterdam, Rotterdam, The Netherlands; 6 Department of Internal Medicine, Erasmus MC, University Medical Center, Room D-411, ‘s Gravendijkwal 230, 3015 CE Rotterdam, The Netherlands

**Keywords:** Transcription factor, Regulatory region, Pituitary, Genetic screening, Growth, Hypopituitarism

## Abstract

**Purpose:**

Mutation frequencies of *PROP1, POU1F1* and *HESX1* in patients with combined pituitary hormone deficiencies (CPHD) vary substantially between populations. They are low in sporadic CPHD patients in Western Europe. However, most clinicians still routinely send DNA of their CPHD patients for genetic screening of these pituitary transcription factors. Before we can recommend against screening of *PROP1, POU1F1* and *HESX1* as part of routine work-up for Western-European sporadic CPHD patients, it is crucial to rule out possible defects in regulatory regions of these genes, which could also disturb the complex process of pituitary organogenesis.

**Methods:**

The regulatory regions of *PROP1, POU1F1* and *HESX1* are not covered by Whole Exome Sequencing as they are largely located outside the coding regions. Therefore, we manually sequenced the regulatory regions, previously defined in the literature, of *PROP1, POU1F1* and *HESX1* among 88 Dutch patients with CPHD. We studied promoter SNPs in relation to phenotypic data.

**Results:**

We found six known SNPs in the *PROP1* promoter. In the *POU1F1* promoter, we found one new variant and two known SNPs. We did not find any variant in the *HESX1* promoter.

**Conclusion:**

Although the new *POU1F1* variant might explain the phenotype of one patient, the general conclusion of this study is that variants in regulatory regions of *PROP1, POU1F1* and *HESX1* are rare in patients with sporadic CPHD in the Netherlands. We recommend that genetic screening of these pituitary transcription factors should no longer be part of routine work-up for Western-European, and especially Dutch, sporadic CPHD patients.

**Electronic supplementary material:**

The online version of this article (10.1007/s11102-017-0850-6) contains supplementary material, which is available to authorized users.

## Introduction

Pituitary hormones are crucial for correct human growth and development. The anterior part of the pituitary (‘master gland’) is the central regulator of peripheral hormones. It contains somatotrope, lactotrope, gonadotrope, thyrotrope and corticotrope cells producing GH, PRL, LH/FSH, TSH and ACTH, respectively. Pituitary embryogenesis is a complex process, which is orchestrated by a number of signaling molecules and transcription factors [1].

During pituitary organogenesis, regulation of the expression of pituitary transcription factors is crucial for normal pituitary development. Transcription factors bind to short DNA sequences usually located in the promoter region of a gene, to guide and activate DNA transcription. Hesx1 is one of the earliest transcription factors that appear in the pituitary gland. As studied in mice, *Hesx1* and *Prop1* exhibit temporally distinct but overlapping patterns of expression over the entire period of pituitary development. They heterodimerize on the same sequence element and Hesx1 suppresses *Prop1* expression (and vice versa). Prop1 has to be present before Pou1f1 (formerly called Pit1) can be activated. Therefore the gene was referred to as Prophet of Pit1. However, *premature* expression of *Prop1* can entirely block pituitary organogenesis [2].

Previous research has shown that bad timing or abnormal amounts of transcription factors can disturb this complex collaboration between transcription factors and lead to abnormal pituitary organogenesis and pituitary hormone deficiencies [3–6]. Since defects in the regulatory regions of a gene can alter expression of the gene, variations in regulatory regions of pituitary transcription factors are likely to also cause isolated or combined pituitary hormone deficiencies. This is supported by the work of Godi et al. [7], who found an association between two polymorphisms within and around a regulatory region in the intron 1 of *PROP1* (rs73346254A and rs148607624 delTAG), and combined pituitary hormone deficiencies (CPHD). Co-presence of the alternative alleles of both SNPs was associated with reduced transcriptional activity as shown by a decreased luciferase activity. An EMSA with oligonucleotide probes carrying the two alternative alleles demonstrated a strong difference in binding specificity of the two allelic sequences for a component present in nuclear extracts from GH4C cells.

**Fig. 1:**
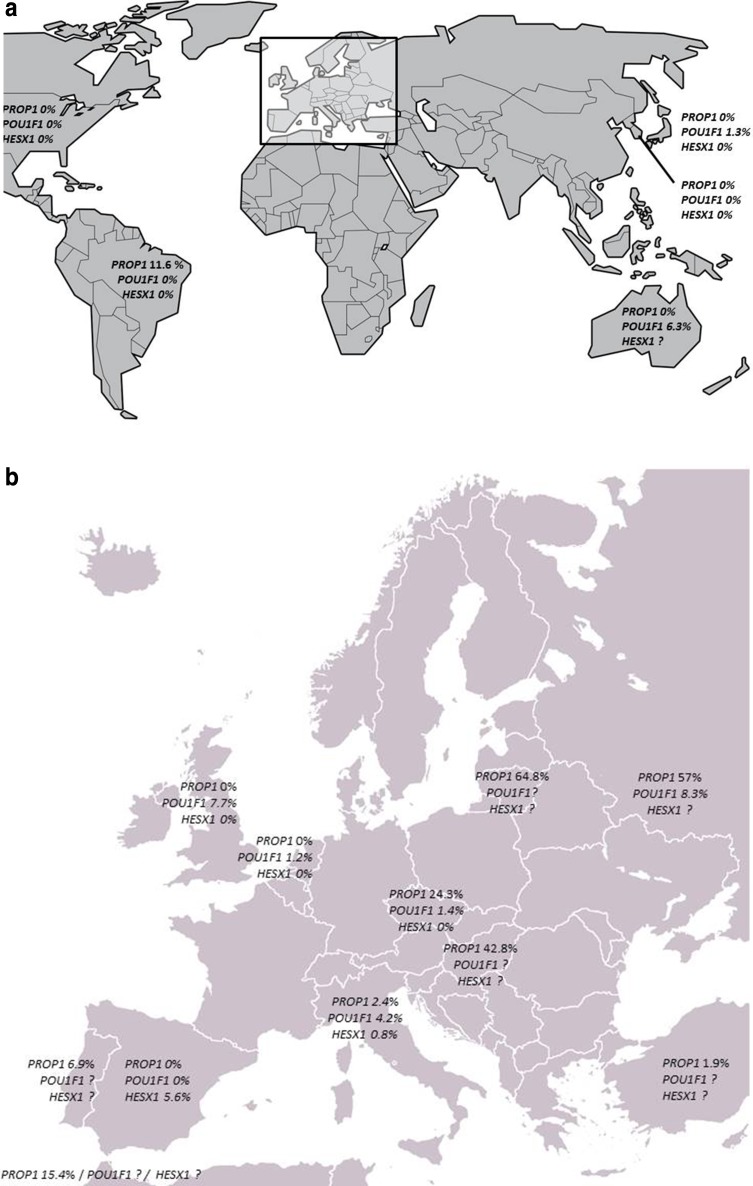
Prevalence of *PROP1, POU1F1* and *HESX1* mutations in CPHD patients as reported in the literature, according to country of origin (see “Methods” section for literature search criteria). Apart from the Moroccan data, most populations included both familial and sporadic CPHD cases. For original data, see Ref [8–20]. ? = no mutation rate available. *The exact mutation frequency in French and German sporadic CPHD patients could not be deduced from the literature [13, 21]

Although our knowledge of pituitary organogenesis and its genetics has increased tremendously in the past two decades, the exact genetic background of CPHD has not yet been clarified. Mutation frequencies reported in the literature vary substantially between populations. Figure 1a, b show the wide range of reported mutation frequencies from various countries, the highest prevalences being present in Eastern Europe.

Therefore, the Dutch national HYPOPIT study was initiated, with the aim to determine the prevalence of genetic defects in the Dutch national cohort of patients with idiopathic pituitary hormone deficiencies.

Within the HYPOPIT study, we previously studied Dutch CPHD cases for mutations in *PROP1, HESX1, POU1F1, LHX3, LHX4, OTX2, SHH* and *HHIP* [22–24] and patients with Isolated GH Deficiency (IGHD) for *GH1, GHRHR, HMGA2* and *CDK6* [25–27]. In the cohort of CPHD patients, we found only one mutation, namely a missense mutation in *POU1F1*. In the IGHD cohort, we found *GH1* mutations in 6% of the participating families, whereas we did not find any mutations in *GHRHR* or the regulatory regions of *GH1*.

This illustrates that only a small minority of the Dutch CPHD and IGHD cases can be explained by genetic defects in coding regions of the genes associated with pituitary hormone deficiencies. This is surprising, because the CPHD and IGHD patients in the Dutch cohort have the same phenotype as patients from other countries, where mutations in *PROP1, POU1F1* and *HESX1* are much more prevalent.

Although the chance of finding a mutation in *PROP1, POU1F1* and *HESX1* in a CPHD patient is very low for Western European countries, especially the Netherlands, most clinicians still routinely send DNA of their CPHD patients for genetic screening of *PROP1, POU1F1* and *HESX1*. However, based on the above mentioned findings, we can conclude that defects in *PROP1, POU1F1* and *HESX1* are usually not the cause of CPHD in Dutch sporadic CPHD patients. It would therefore make sense to recommend against screening of *PROP1, POU1F1* and *HESX1* as part of routine work-up for Dutch sporadic CPHD patients. However, in order to justify such a recommendation, defects in the regulatory regions of these genes should first be ruled out.

The regulatory regions of *PROP1, POU1F1* and *HESX1* are largely located outside the coding regions and are thus not covered by Whole Exome Sequencing. Therefore, in the current study, we manually sequenced regulatory regions of *PROP1, POU1F1* and *HESX1* among 88 patients with CPHD. Since patients with *HESX1* mutations can initially present with IGHD only, we also screened 92 patients with IGHD. We compared genotype frequencies in our CPHD cohort with those found in healthy controls from the 1000 genomes database and we studied promoter SNPs in relation to phenotypic data.

### Patients and methods

#### Patients

Clinical data of the patients were collected from the Dutch National Registry of Growth Hormone Treatment, where clinical and laboratory parameters of GH-treated Dutch patients are being registered. We included all GH-treated children and adults registered in the Dutch National Registry of Growth Hormone Treatment between 1992 and 2003, who had deficiencies of GH and one or more additional hormonal axes and were treated in the hospitals participating in the study. Patients with Growth Hormone deficiency (GHD) of known cause, such as a brain tumour, brain surgery, brain irradiation and known syndromes, were excluded. GHD was defined as a peak GH response below 6.7 μg/L (20 mU/L) to arginine or clonidine test, or 10 μg/L (< 30 mU/L) combined with serum IGF-I < − 2 SDS. Patients were classified as having severe IGHD or partial IGHD (pIGHD) based on a scoring system including height SDS (HSDS), maximum stimulated GH levels, IGF-I SDS and IGFBP-3 SDS. Central hypothyroidism was defined as abnormal TRH test or TSH levels that were low or inadequate for low (F) T4, using reference ranges of each participating hospital. Central hypocortisolism was defined as abnormal CRF/ACTH/glucagon test or ACTH levels which were low or inadequate for low cortisol. Central hypogonadism was diagnosed when LH, FSH and estrogen/testosterone or LHRH test were low for age, or when spontaneous puberty had not occurred at age 14 years. Prolactin deficiency was defined as abnormal random prolactin or during TRH testing. For all hormone values, reference values of the participating hospitals are available upon request.

The Medical Ethics Committees of all participating hospitals approved the study. Informed consent was obtained from all participating patients and their parents, if patients were younger than 18 years.

#### Methods

##### PCR

**Fig. 2:**
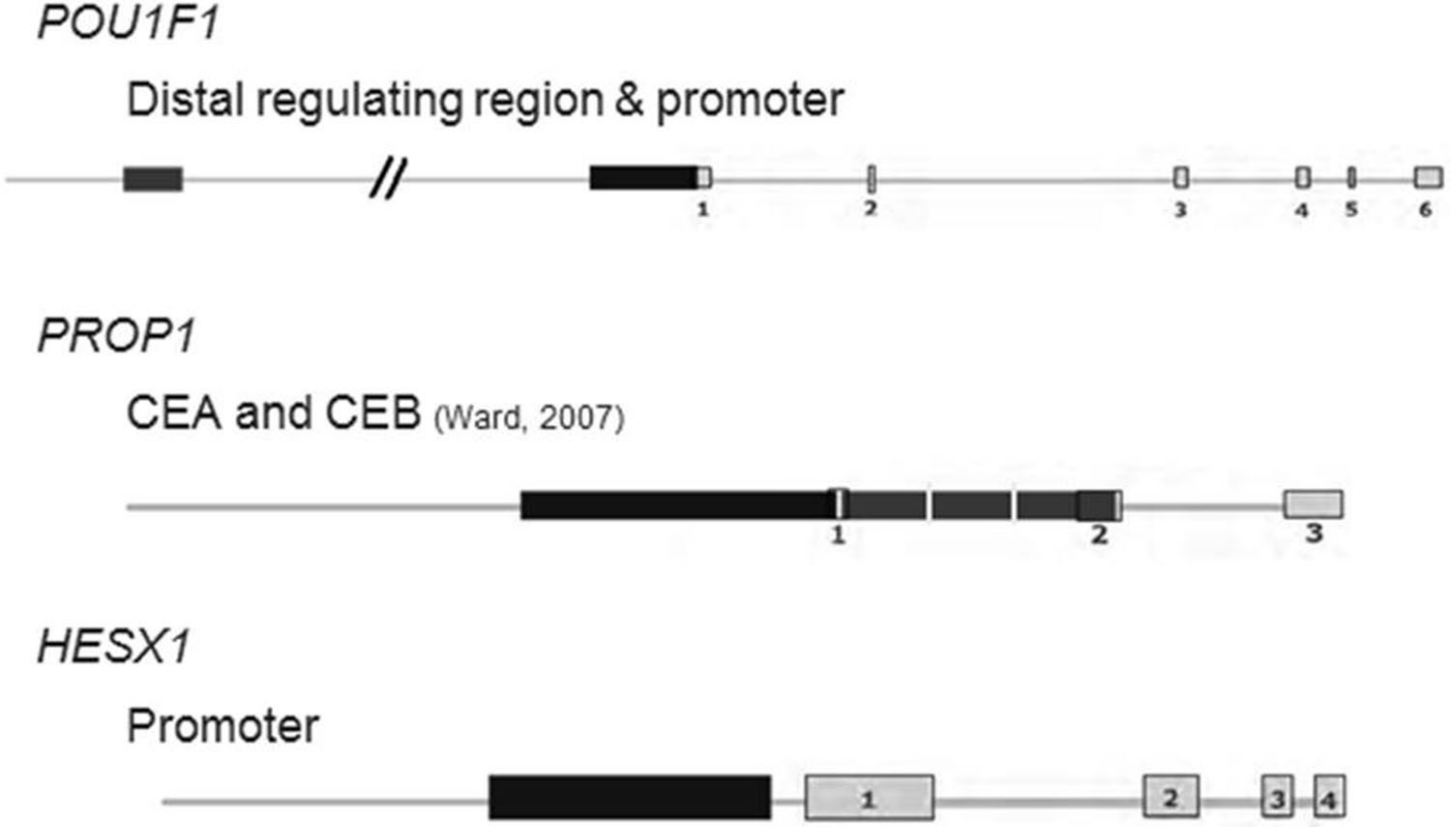
Overview of regulatory regions of *PROP1, POU1F1* and *HESX1. CEA* conserved element A, *CEB* conserved element B

Genomic DNA was extracted from samples of peripheral venous blood according to standard procedures. For PCR, we used the following Qiagen reagents: 10× PCR buffer, 0.2 mM dNTP mixture and 5 units/μL Taq DNA Polymerase. After gel electrophoresis, we purified the PCR products using the High Pure PCR product Purification kit (QIAGEN^®^, Hilgen, Germany) or the Illustra GFX 96 PCR Purification Kit (GE Healthcare^®^, Leiderdorp, the Netherlands) according to the supplied protocols. PCR conditions and amplification programs are available upon request. We studied the regulatory regions of *POU1F1* as described by Delhase et al. [28] and the promoter of *HESX1* as described by Eroshkin et al. [29]. Primer sequences were designed using the Primer3 program. Nucleotide sequences were obtained from Ensembl (*PROP1*: ENSG00000175325; *POU1F1* ENSG00000064835; *HESX1* ENSG00000163666). Primer sequences for CEA and CEB of *PROP1* were kindly provided by Godi et al. [7] and Ward et al. [30]. An overview of the regulatory regions of *PROP1, POU1F1* and *HESX1* is shown in Fig. 2. The primers were ordered from Integrated DNA Technologies Inc (Iowa, United States), dissolved in sterile H_2_O to a concentration of 50 pmol/μL and stored at − 20 °C until further use. From this stock, aliquots with a working concentration of 25 pmol/μL were made and stored at − 20 °C.

##### Sequencing

Sequencing was outsourced to Baseclear, a commercial DNA sequencing company in Leiden, the Netherlands, and carried out using an ABI3730XL sequencer. The results were analyzed for mutations using Sequencher4.1 (Genecodes, Michigan, United States).

##### Statistics

We analysed clinical and genotypic data by means of ANOVA and Chi square tests using SPSS version 22.0.

##### Literature

In order to make an overview of *PROP1* mutation frequencies according to country of origin, we performed a pubmed search using ‘*PROP1* AND mutation AND combined pituitary hormone deficiency’ (filter: English). We only selected articles of which the full text manuscript was available online or sent to us by the authors upon request. Articles in which the country of origin of the patients was mixed or unclear, were excluded.

##### In silico analysis of new variant

We used online software PROMO 3.0 [31] to predict changes in transcription factor binding sites in the *POU1F1* promoter region.

## Results

We included 88 CPHD patients (63 M/25 F) and 92 patients (64 M/28 F) with IGHD, either classified as severe (‘IGHD’) or partial IGHD (‘pIGHD’). The male predominance in our cohort is in accordance with the male predominance in the overall Dutch CPHD and IGHD population.

Clinical characteristics of the CPHD and IGHD patients are shown in Table 1. All CPHD patients had at least GHD and thyroid hormone replacement, while 79% of the patients additionally required glucocorticoid hormone replacement and 85% required induction of puberty. Hypoprolactinaemia was present in only 15% of CPHD patients tested and four patients (5%) had diabetes insipidus. Neonatal jaundice, neonatal hypoglycaemia and micropenis, suggestive of early pituitary hormone deficiencies, were present in 62, 43 and 38% (of boys), respectively. 79 patients underwent MRI, 82% of them had an abnormal pituitary on MRI (Figure S1). Clinical characteristics of the (p)IGHD patients screened for defects in the *HESX1* promoter are also shown in Table 1.

**Table 1:** Clinical characteristics of the CPHD and (p)IGHD patients in this study

	CPHD	(p)IGHD
Sex	63 M/25 F	64 M/28 F
Birth weight (kg) (SDS)	3.0 (0.8) − 0.6 (1.4)	3.1 (0.6) − 0.7 (1.3)
Birth length (kg) (SDS)	49.4 (3.0) − 0.3 (1.4)	48.1 (3.6) − 0.7 (1.3)
Gestational age (w)	38.9 (2.8)	38.9 (2.6)
Neonatal jaundice	62%	21%
Neonatal hypoglycaemia	43%	10%
Micropenis (% of boys)	38%	17%
Age at start of GH treatment (y)	4.0 (3.5)	6.4 (3.4)
Height SDS at start of GH	− 3.0 (1.3)	− 3.2 (0.9)
GH peak during Arginine test (μg/L)	1.7 (2.0)	3.1 (2.0)
GH peak during Clonidine test (μg/L)	1.5 (1.3)	3.7 (2.0)
IGF-I SDS	− 4.4 (3.0)	− 3.3 (2.4)
Thyroid hormone replacement	100%	14%^a^
Hydrocortison treatment	79%	0
Induction of puberty	85%	0
Hypoprolactinemia	15%	0
Diabetes insipidus	5%	0
Affected first-degree relatives	5%	7%

Data are expressed as mean (SD) or %

*SDS* standard deviation score

^a^No cases of central hypothyroidism, only primary hypothyroidism or borderline low FT4 values during GH therapy

We identified nine genetic variants in the CPHD cohort; six in the regulatory regions of *PROP1* and three in the regulatory regions of *POU1F1* (Table 2). We did not find any variants in the *HESX1* promoter in either the (p)IGHD or CPHD patients. Four of the *PROP1* promoter SNPs have been previously reported by Godi et al. [7]. The genotype frequencies did not differ significantly from the frequencies found in healthy controls from the 1000 Genome Project (Table 2).

**Table 2:** New and known variants in the regulatory regions of *PROP1, POU1F1* and *HESX1* in the current study, compared to British control data from the 1000 genomes database

Gene	rs number	Frequency (%) in current study/British controls* (p)
*POU1F1* promoter	New variant (− 1295C > T)	1.1/– (NS)
*POU1F1* promoter	rs10511134 (T > A)	43.8/50.5 (NS)
*POU1F1* promoter	rs300982 (C > T)	10.9/3.8 (NS)
*POU1F1* DRR	No variants found	–
*PROP1* CEA	rs12654239 (G > T)	61.4/53.3 (NS)
*PROP1* CEB	rs4431364 (C > G)	28.9/31.9 (NS)
*PROP1* CEB	rs4498267 (C > T)	12.5/13.2 (NS)
*PROP1* CEB	rs148607624 (ins/delTAG)	1.7/0.5 (NS)
*PROP1* CEB	rs141213827 (delG)	1.1/0 (NS)
*PROP1* CEB	rs113776172 (C > A)	1.1/0 (NS)
*HESX1* promoter	No variants found	–

*NS* not significant

A polymorphism in *PROP1*, consisting of a 3 bp (TAG) deletion in the promoter (ins/delTAG, rs148607624), which was previously shown to be more frequent among CPHD patients than among controls [7], was associated with a slightly more severe phenotype (i.e. shorter height, lower peak GH during GH test, and lower serum IGF-I SD scores) in the current cohort (Table 3). The other SNPs were not related to phenotype.

**Table 3:** Severity of GHD phenotype according to *PROP1* SNP rs148607624 (ins/delTAG)

	N	Mean	SD	Min	Max	p
Height at start of GH treatment						
TAG/TAG	84	− 3.1	1.3	− 6.0	0.0	0.22
–/TAG	2^a^	− 4.2	0.4	− 4.5	− 4.0	
Mean of peak GH values during clonidine and arginine tests (μg/L)						
TAG/TAG	53	1.8	2.1	0.0	9.4	0.49
–/TAG	3	0.9	0.9	0.0	1.7	
IGF-I SDS at start of GH treatment						
TAG/TAG	57	− 3.8	2.5	− 9.4	1.4	0.010
–/TAG	3	− 7.8	2.1	− 9.0	− 5.3	

^a^Numbers do not add up to 88, because height SDS at start GH was not available for two patients. In these two patients, GH was started based on deficiencies of all other pituitary axes, low IGF-I SDS and two severely deficient GH tests

One of the *POU1F1* variants had not been reported in the 1000 Genomes database by January 2017. We found this new heterozygous variant, − 1295C > T in a girl born Small for Gestational Age (SGA) (2500 g and 45 cm after 41 w of gestation). At 6 months of age, her length was − 4.3 SDS. After endocrine function tests, she was diagnosed with GH deficiency and later with central hypothyroidism. She also required hydrocortisone treatment and sex steroid treatment due to lack of pubertal development. Her MRI revealed no visible abnormalities.

To predict the possible impact of this new *POU1F1* promoter variant, we performed in silico analysis using online software. The binding sites for several transcription factors disappear when the C located 1295 bp upstream of the transcription start site is replaced by a T. In addition, the new variant creates new binding sites for other transcription factors, shown in Supplementary table S1, which means that transcription of *POU1F1* might be affected by this variant.

## Discussion

We studied the regulatory regions of the pituitary transcription factors *PROP1, POU1F1* and *HESX1* in a cohort of 88 CPHD patients, in order to find an explanation for their phenotype. Since patients with *HESX1* mutations can initially present with IGHD only, we also screened 92 patients with severe or partial IGHD (64 M/28 F).

In the CPHD patients, we identified eight known polymorphisms (six in the regulatory regions of *PROP1* and two in the regulatory regions of *POU1F1*) and one new *POU1F1* variant. No variants in the *HESX1* promoter were identified in the 88 CPHD and 92 (p)IGHD patients.

Since CPHD can be caused by both heterozygous and homozygous *POU1F1* mutations, the new heterozygous *POU1F1* promoter variant − 1295 C > T could be the cause of CPHD in this patient. We performed in silico analysis to predict its possible impact, which showed that the − 1295 C > T variant might create new binding sites for several transcription factors in the *POU1F1* promoter region. In theory, this could increase or decrease the activity of the promoter, and therefore alter the expression of *POU1F1*. However, even though this new variant might explain the phenotype of this individual patient, the general conclusion of this study is that variants in regulatory regions of *PROP1, POU1F1* and *HESX1* are rare in patients with sporadic CPHD in the Netherlands.

These results are very important for clinicians and researchers in the field of congenital hypopituitarism. Most clinicians routinely send DNA of their familial and sporadic CPHD patients to genetic laboratories for genetic screening of *PROP1, POU1F1* and *HESX1*. However, the chance of finding a mutation in these genes in a CPHD patient depends strongly on the country of origin and is very low for Western European countries, especially for the Netherlands. Our previous study already showed that the vast majority of Dutch sporadic CPHD cases could not be explained by defects in the coding regions of *PROP1, POU1F1* and *HESX1* [24]. The results of the current study show that defects in their regulatory regions do not explain the phenotype either.

Based on the current study and our previous studies investigating pituitary transcription factors, we therefore recommend that genetic screening of *PROP1, POU1F1* and *HESX1* should no longer be part of routine work-up for sporadic CPHD patients in the Netherlands.

The fact that we studied a Dutch cohort, suggests that our results only justify recommendations for Dutch patients. However, similar recommendations might also apply to other Western European countries, because of our shared genetic background. The genetic composition of European countries is in part determined by human migration in the Paleolithic and Neolithic periods, but also by more recent migration in the fifteenth and seventeenth century. The genetic background seems to be comparable for most Western European countries, except for the Iberian Peninsula. This has been confirmed by recent research concerning *PROP1* mutation frequencies in different countries [32, 33]. *PROP1* mutation rates are highest in Russian, Eastern European, Portuguese and Brazilian cohorts. In Western and Southern European Countries, *PROP1* frequencies among sporadic CPHD patients are generally low (United Kingdom 0%, Spain 5.6%, Portugal 6.9%, Italy 2.9%, Fig. 1b). De Rienzo et al. [33] state that after excluding Russian, Eastern European, Portuguese and Brazilian cohorts, the global frequency of *PROP1* mutation decreases from 11.2 to 5.1% (a decrease from 6·7 to 1·9% in sporadic patients and 48.5–35.8% in familial cases). The mutation prevalence found in the current Dutch cohort is among the lowest in Europe.

In the case of sporadic CPHD, mutations are only likely to be identified in patients from countries with a high prevalence of mutations and patients who present with a phenotype that strongly favours the analysis of a particular gene, such as *LHX3* (hypopituitarism, sensorineural hearing loss and skeletal abnormalities) or *HESX1* (hypopituitarism and SOD) [33]. Parents of sporadic CPHD patients without these specific phenotypes should be informed about these low mutation rates in order to prevent false expectations.

In contrast to sporadic cases, familial subjects from all countries should undergo genetic analysis if possible. In these familial cases, the likelihood of detecting a mutation within any of the transcription factor genes is much higher [10–16, 19, 20, 33].

In conclusion, mutations in the regulatory regions of pituitary transcription factors *PROP1, POU1F1* and *HESX1* are rare in Western Europe, especially in the Netherlands. Based on this and previous studies of our group, and based on mutation frequencies from other countries, we conclude that these mutations do not significantly contribute to the prevalence of IGHD or CPHD. We therefore recommend against routine genetic screening of *PROP1, POU1F1* and *HESX1* among sporadic CPHD patients in Western Europe, especially in the Netherlands. Further research should focus on the identification of new candidate genes, using a whole genome/exome approach.

## Electronic supplementary material

Below is the link to the electronic supplementary material.

Supplementary material 1 (DOCX 70 KB)

## Abbreviations

ACTH: Adrenocorticotropic hormone
CEA: Conserved element A
CEB: Conserved element B
CPHD: Combined pituitary hormone deficiency
DRR: Distal regulating region
FSH: Follicle stimulating hormone
GH: Growth hormone
GHD: Growth hormone deficiency
GHRH: Growth hormone releasing hormone (GHRF)
*HESX1*: Homeobox expressed in ES cells 1
(p)IGHD: (partial) Isolated growth hormone deficiency
LH: Luteinising hormone
PCR: Polymerase chain reaction
*POU1F1*: POU domain, class 1, transcription factor 1 (PIT1)
PRL: Prolactin
*PROP1*: Prophet of *POU1F1*
SNP: Single nucleotide polymorphism
SOD: Septo-optic dysplasia
T3/T4: Thyroid hormone
TRH: Thyrotropin-releasing hormone
TSH: Thyroid stimulating hormone
