# Supplementary material for: Genetic screening of regulatory regions of pituitary transcription factors in patients with idiopathic pituitary hormone deficiencies
Source: Pituitary. 2017 Dec 18;21(1):76–83. doi: 10.1007/s11102-017-0850-6 (PMC5767207; doi:10.1007/s11102-017-0850-6)
Supplement: Supplementary file 1 — Supplementary material 1 (DOCX 70 KB) [file 11102_2017_850_MOESM1_ESM.docx]

**Supplementary data**

**Table S1** Transcription factors expressed in human pituitary, for which binding sites are located in the region surrounding the new *POU1F1* promoter variant-1295 C>T.

^1^ Existing binding site disappears in case the new variant -1295C>T is present, ^2^ New binding site appears in case the new variant -1295 C>T is present

| **NT at -1295** | **Transcription factor** |
| --- | --- |
| C^1^ | c-Fos, c-Jun, LCR-F1, NHP-1, Pax-2, RORα2, TGIF, USF2b |
| C/T | FOXP3, POU2F2 |
| T^2^ | aMEF2, GRα, HNF-3α |

***
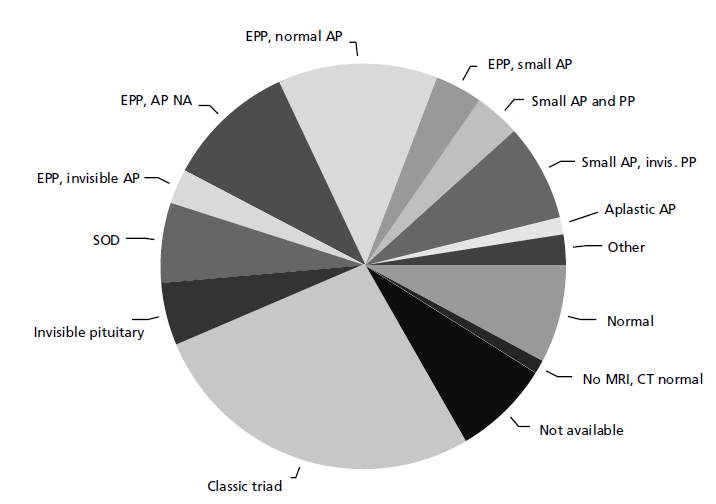
***

**Figure S1**

MRI data of CPHD patients participating in the current study. AP = anterior pituitary, PP = posterior pituitary, EPP = ectopic posterior pituitary, SOD = Septo-Optic Dysplasia, classic triad = hypoplastic anterior pituitary, ectopic posterior pituitary and absent or interrupted pituitary stalk
